# Insights from Molecular Dynamics Simulations: Structural Basis for the V567D Mutation-Induced Instability of Zebrafish Alpha-Dystroglycan and Comparison with the Murine Model

**DOI:** 10.1371/journal.pone.0103866

**Published:** 2014-07-31

**Authors:** Davide Pirolli, Francesca Sciandra, Manuela Bozzi, Bruno Giardina, Andrea Brancaccio, Maria Cristina De Rosa

**Affiliations:** 1 Istituto di Biochimica e Biochimica Clinica, Università Cattolica del Sacro Cuore, Rome, Italy; 2 Istituto di Chimica del Riconoscimento Molecolare (ICRM) - CNR c/o Università Cattolica del Sacro Cuore, Rome, Italy; Jacobs University Bremen, Germany

## Abstract

A missense amino acid mutation of valine to aspartic acid in 567 position of alpha-dystroglycan (DG), identified in dag1-mutated zebrafish, results in a reduced transcription and a complete absence of the protein. Lacking experimental structural data for zebrafish DG domains, the detailed mechanism for the observed mutation-induced destabilization of the DG complex and membrane damage, remained unclear. With the aim to contribute to a better clarification of the structure-function relationships featuring the DG complex, three-dimensional structural models of wild-type and mutant (V567D) C-terminal domain of alpha-DG from zebrafish were constructed by a template-based modelling approach. We then ran extensive molecular dynamics (MD) simulations to reveal the structural and dynamic properties of the C-terminal domain and to evaluate the effect of the single mutation on alpha-DG stability. A comparative study has been also carried out on our previously generated model of murine alpha-DG C-terminal domain including the I591D mutation, which is topologically equivalent to the V567D mutation found in zebrafish. Trajectories from MD simulations were analyzed in detail, revealing extensive structural disorder involving multiple beta-strands in the mutated variant of the zebrafish protein whereas local effects have been detected in the murine protein. A biochemical analysis of the murine alpha-DG mutant I591D confirmed a pronounced instability of the protein. Taken together, the computational and biochemical analysis suggest that the V567D/I591D mutation, belonging to the G beta-strand, plays a key role in inducing a destabilization of the alpha-DG C-terminal Ig-like domain that could possibly affect and propagate to the entire DG complex. The structural features herein identified may be of crucial help to understand the molecular basis of primary dystroglycanopathies.

## Introduction

Dystroglycan (DG) is a pivotal member of the dystrophin-glycoprotein complex (DGC), which links the cytoskeleton to the extracellular matrix (ECM) via dystrophin [1]. Essential for normal muscle function, DG also has important roles in a wide range of tissues, including central and peripheral nervous systems, and in the maintenance of epithelial structures [2]. DG is synthesized as a precursor protein that is post-translationally cleaved into the α- and β- subunits. Within the DGC, the α-subunit is located outside the plasma membrane and binds ECM proteins, such as laminin and agrin. α-DG is extensively glycosylated and its correct glycosylation is essential to elicit its ligand binding activity [3]. Mutations in a growing number of genes encoding for glycosyltransferases or associated proteins involved in DG glycosylation give rise to a class of congenital as well as limb-girdle muscular dystrophies, which are known as secondary dystroglycanopathies [4], [5]. It is worthwhile to notice that, to date, only two patients affected by recessive primary dystroglycanopathies, associated with mutations in the DG encoding gene *DAG1* (c.575C>T, T192M and c.2006G>T, C669F) have been described [6], [7].

The importance of the DG gene for muscle stability has been confirmed also in zebrafish (*Danio rerio*) [8], an organism that represents a reliable model for human muscular diseases [9]–[12] and that is frequently employed for investigating the effect of drugs alleviating the symptoms of Duchenne muscular dystrophy [13]–[15].

**Figure 1:**
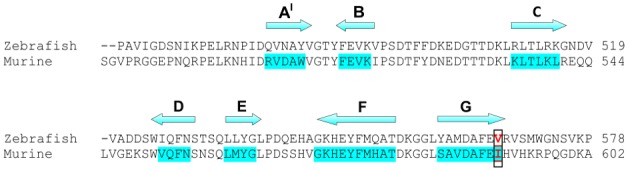
Amino acid sequence alignment of zebrafish and murine Ig-like domains belonging to the α-DG C-terminal region. The alignment reflects the equivalence of residues in the two structures. At the top is shown the location of the strands predicted by our molecular model of murine α-DG [17]. The positions of point mutations (V567D and I591D for zebrafish and murine DG, respectively) are shown in red.

Recently, in an attempt to identify novel genes responsible for skeletal muscle disorders, a zebrafish mutant was identified that showed impaired locomotion behavior and dystrophic muscles [16]. Such point mutation (c.1700T>A) in *DAG1*, resulting in a missense mutation V567D, induced destabilization of the DG complex and membrane damage. In particular, genetic and biochemical studies showed that the V567D substitution is associated with a strong reduction of DG transcripts and a complete absence of α and β subunits [16]. However, despite the experimental characterization of many functional effects of the V567D substitution in α-DG, a detailed molecular framework explaining the observed destabilization and loss-of-function is still lacking. Comprehensive details at atomic resolution about the structural perturbations induced by the V567D substitution thus remain elusive. For this reason, and given our experience with these systems, which led us to identify a second immunoglobulin-like (Ig-like) domain in murine α-DG C-terminal region [17] and ε-sarcoglycan [18], we have exploited the capabilities of molecular dynamics (MD) simulation to investigate the structural and dynamical changes of zebrafish α-DG caused by V567D replacement. In fact, we have recently predicted and then experimentally demonstrated using recombinant proteins that not only residues 60–158 of murine α-DG display an Ig-like β-sandwich fold [19], but also residues ranging from ∼500 to 600 [17]. We showed that the murine α-DG C-terminal is a typical β-sandwich Ig fold consisting of two β-sheets forming a β-sandwich. The first sheet contains three anti-parallel strands (B, E, D), whereas the second sheet comprises strands A′, G, F and C, with A′ packing parallel with the C terminus of strand G, the others arranged in an anti-parallel fashion [20]. The zebrafish V567D substitution [16] falls within a region of α-DG which has proved to be of crucial importance to understand the role of the Ig-like domain in the interaction with the extracellular N-terminal domain of β-DG [17], [21]. Gupta et al. [16], using a number of algorithms able to predict whether an amino acid substitution affects protein function, hypothesized that the V567D mutation deeply compromises protein function resulting in a pathological phenotype. Nevertheless, the structural role of this mutation remains unclear. To fill this gap, here we investigated the effects of V567D mutation on the zebrafish α-DG structure through molecular dynamics (MD) simulations, which allow the study of the conformational characteristics of the protein at every step during the computational simulations [22]. Subsequent structural *in silico* analyses were performed. Exploiting our murine α-DG model, we also examined the structural effects of the mutation I591D, which is topologically equivalent to the V567D mutation (Fig. 1), combining computational and biochemical analysis.

The present MD studies revealed that the conformational stability of mutated DG is considerably reduced compared to wild-type, with a significant breakdown in the secondary structure observed for zebrafish V567D. Potential implications in processes leading to dystroglycanopathies are discussed.

## Materials and Methods

### Constructing structural models of wild-type and mutant α-DG C-terminal domains

Following the same procedure used in De Rosa et al. [17] the theoretical atomic models of wild-type and mutated α-DG C-terminal regions (residues 462–626 and 483–651 for zebrafish and murine proteins, respectively) were constructed using the I-TASSER server [23], [24]. Starting from the original sequence of wild-type protein retrieved from the UniProt Database [25] (accession numbers Q499B9 and Q62165 for zebrafish and murine DG, respectively), the first step I-TASSER performed was to create a sequence profile for the query using PSI-BLAST [26]. The secondary structure of each of these sequences was then predicted using PSIPRED [27], a highly accurate secondary structure prediction server (http://bioinf.cs.ucl.ac.uk/psipred). Using the constraints provided by PSI-BLAST and PSIPRED, the query was then threaded through the PDB structure library using the Local Meta-Threading-Server (LOMETS) [28], which uses eight servers to find the best possible templates for the query. The continuous fragments from the threading alignments were then excised from their respective template structures and assembled into a full-length model, whereas the unmatched regions were built via *ab initio* modelling. Hence, unlike other homology modelling software, this server predicts the structure even when there are no matched sequences in known PDB structures. The quality of each predicted structure was assessed with a scoring method, and five atomistic models with the highest scores were obtained for each input protein sequence. The best models among those predicted by I-TASSER were checked using the programs PROCHECK [29], VERIFY3D [30] and ProSA-Web (https://prosa.services.came.sbg.ac.at/prosa.php) [31]. Visualization and molecular graphics were performed using Discovery Studio (Accelrys Inc.) on the workstation HP XW8600 running Red Hat Enterprice Linux 5.

### Molecular Dynamics Simulations and Analysis

The best-scored wild-type and mutant DG model structures obtained by I-TASSER were chosen as the starting coordinates for the MD simulation. Calculations were performed as reported earlier [17], with slight modifications of the size and shape of the simulation box. Briefly, all simulations were carried out using the 4.5.1 version of GROMACS [32] and GROMOS96 force field [33]. Each structure was immersed in a triclinic box with periodic boundary conditions. The SPC water model was used [34] and the systems were neutralized by 2 and 3 Na^+^ ions (wild-type and mutant zebrafish, respectively) and by 3 and 2 Cl^−^ ions (wild-type and mutant murine, respectively). The box dimensions (7.3 nm×5.6 nm×8.5 nm and 6.9 nm×7.1 nm×9.6 nm for zebrafish and murine, respectively) were set to allow at least 0.9 nm between the protein and the box faces on each side. The final zebrafish systems consisted of 1671 (wild-type) and 1672 (V567D) protein atoms surrounded by 10725 and 10223 water molecules, respectively, whereas the final murine systems consisted of 1712 protein atoms (both wild-type and I591D) surrounded by 14999 and 14980 water molecules, respectively. All the MD simulations were carried out using periodic boundary conditions. The geometry of each system was initially optimized using the steepest descent algorithm and then equilibrated for 20 ps. Next, the molecular dynamics were run for 40 ns at 300 K, and the data were collected every 5 ps. Constant temperature (300 K, τT = 0.1 ps) was maintained by coupling to a bath using a ν-rescale algorithm [35], whereas pressure was kept at 1 atm using the Parrinello-Rahman barostat [36]. Long range electrostatic interactions were calculated using the Particle-Mesh Ewald Method [37], whereas application of the Lincs method [38] allowed for an integration step size of 2 fs. Two additional replicate simulations with a duration of 40 ns were also performed for each of the systems studied, with differing initial velocities. Analysis of the trajectories was performed using the GROMACS tools g_rms, g_rmsf, g_hbond, g_gyrate and g_sasa. Secondary structure was calculated using the DSSP algorithm [39] within GROMACS.

### DNA manipulations

The single point mutation I591D was introduced into the murine DG construct containing a myc-tag inserted within the C-terminus of α-DG and cloned in pEGFP vector [40]. The I591D mutation was also introduced in a DNA construct encoding for the α-DG C-terminal domain, α-DG(485–630), cloned into the pHis-Trx vector; in both cases, the QuikChange site-directed mutagenesis kit (Stratagene) was used to introduce a mutated triplet (underlined), corresponding to an Asp residue, exploiting the following primers: I593D_S 5′-GTG GAT GCC TTC GAG GAC CAT GTT CAC AAG CGC-3′and I593D_AS 5′-GCG CTT GTG AAC ATG GTC CTC GAA GGC ATC CAC-3′. All constructs were verified by automated sequencing.

### Recombinant expression and purification of α-DG(485–630)I591D

The recombinant fusion protein, 6His-Txr-α-DG(485–630), was expressed in *E.coli* BL21(DE3) Codon Plus RIL strain and purified using nickel affinity chromatography as described elsewhere [21]. The protein of interest, α-DG(485–630), was obtained upon thrombin cleavage. Tricine/SDS-PAGE was used to check the purity of the recombinant protein under analysis.

### Cell culture, transfection and immunoprecipitation

293-Ebna cells, grown in DMEM supplemented with antibiotics and 10% (v/v) fetal calf serum, were transfected with 20 µg of wild-type or I591D DG constructs using the calcium phosphate method as described elsewhere [40]. After 24 h, cells were dissolved in PBS containing 1% Triton X-100 and protease inhibitors (Roche, Switzerland). Cell lysate was resolved on a 10% SDS-PAGE. For Western blot analysis, proteins were transferred to nitrocellulose and probed with an anti β-DG antibody (43-DAG) (1∶50) and with a peroxidase-conjugated secondary antibody (Sigma, USA) diluted 1∶7000 (anti-mouse); the reactive products were revealed using the luminol-based ECL system (Pierce, USA).

All the steps required for immunoprecipitation were carried out using the µMACS Epitope Tag Protein Isolation Kit (Miltenyi Biotec., Germany), following the manufacturer’s instructions. Briefly, 1 ml of total protein extract of transfected cells was incubated with 50 µl of magnetic beads conjugated with an anti-myc antibody (Miltenyi Biotec., Germany) for 30 min in ice. After several washes, the adsorbed protein was eluted with 50 µl of sample buffer and run on a 10% SDS-PAGE followed by Western blot analysis with an anti-myc antibody-HRP conjugated (Miltenyi Biotec., Germany).

## Results and Discussion

The I-TASSER approach has a high success rate to construct correct folds for medium-to-large sized proteins by structurally reassembling the fragments excised from threading template structures without using homologous templates, as demonstrated by the recent CASP experiments [41], [42]. In addition, we recently demonstrated the ability of the I-TASSER server to predict a reliable model of the murine α-DG C-terminal region [17]. The I-TASSER approach was therefore used to find out the secondary and tertiary structures of zebrafish wild-type and V567D α-DG C-terminus, in comparison with the murine α-DG carrying the topologically equivalent mutation I591D. Comprehensive details at atomic resolution concerning the structural perturbations induced by these amino acid substitutions were then obtained by three statistically independent MD simulations, which allowed to refine the predicted structures within a nanosecond time scale and to investigate the conformational changes, occurring upon mutation. In total, 12 MD simulations were performed as 40 ns triplicates for zebrafish wild-type, zebrafish V567D mutant, murine wild-type and murine I591D mutant. The resulting findings are very similar for each set of the three runs and here we report the results of one of the three simulations. The average properties for the three simulations, as expected similar to those of the independent simulations are reported in Figures S2, S3, S4, S5, S6. A graphical representation of secondary structure analysis for the three distinct MD simulations is shown (Fig. S4).

### *In silico* modelling of zebrafish wild-type and V567D α-DG C-terminal region

**Figure 2:**
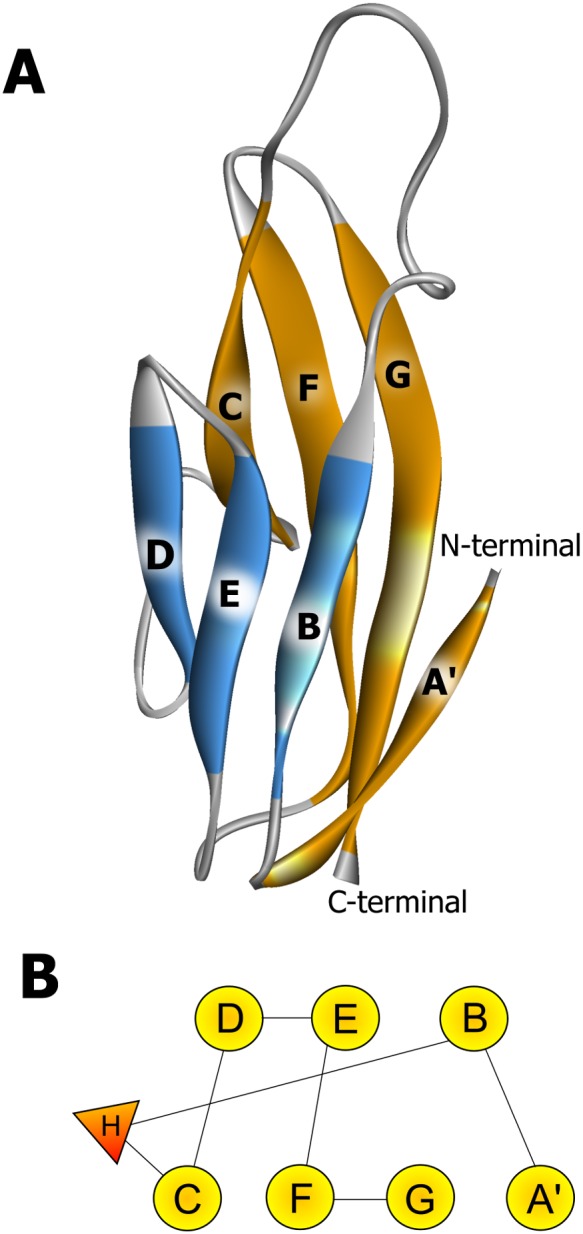
Structure and topology of wild-type and mutant zebrafish Ig-like domains belonging to the α-DG C-terminal region. The secondary structure elements (panel A) are named according to Harpaz and Chothia [46]. The β-strands are colored according to the sheet to which they belong and the N and C termini are indicated. The topology diagram of the domains is shown in panel B; β-strands are shown as circles and the small helix as a triangle.

In both systems, only β-strands and coils were found in the 475–574 region of α-DG, whereas coils, α helices and strands were found in the extreme C-terminus. Figure S1 shows the predicted secondary structures of the two systems. Not surprisingly, the I-TASSER threading procedure identified immunoglobulin-like domains as the best templates for wild-type, specifically, 1U2C (α-DG N-terminal region [19]), 2WCP (mouse chaderin-23 [43]), 2YST (human protocadherin 7, to be published) and 3Q2V (mouse E-cadherin ectodomain [44]). The same templates, with the exception of 2YST were identified for the mutant V567D. The overall sequence identity shared between the α-DG C-terminal regions and each of the templates is approximately 24%. Although this value is quite low, it is similar to other cases in which modelling has been applied [45]. The quality of the generated models was assessed in I-TASSER based on two major criteria, the C- and the TM-scores. The C-score is calculated based on the significance of the threading alignments and the convergence of the I-TASSER simulations. C-scores typically range from −5 to 2, with higher scores reflecting a model of better quality. The TM-score is a measure of structural similarity between the predicted model and the native or experimentally determined structure, with a value >0.5 indicating a model of correct topology. Assessments for the zebrafish α-DG C-terminal regions are reported in Table 1 indicating reasonable models and accurate topology. In all cases, search of the PDB, quantified by TM-score, indicated 1U2C as the structure with the highest structural similarity (Table 1). As expected, the zebrafish α-DG model, as well as that of the V567D mutant, is similar in structure to the murine α-DG [17] with a root mean square deviation (RMSD) of the Cα atoms of 1.92Å (wild-type) and 1.68Å (V567D). According to I-TASSER then, the region encompassing residues 475–574 of the α-DG C-terminus adopts the typical I-frame immunoglobulin superfamily fold and is stabilized by extensive hydrophobic core interactions between the two β-sheets [46] (Fig. 2). Consistently with the 1U2C structure and our previous results, a small helix was detected between β-strands B and C (residues 495–498). The rest of the region (residues 575–626) displayed two coil-strand-coil regions separated by a helix.

**Table 1:** Statistical support for the predicted structural models obtained from I-TASSER.

α-DG	Quality of Predicted Model	
	C-score	TM-Score^a^
Wild-type zebrafish	−0.8	0.8
V567D zebrafish	−0.9	0.8
Wild type murine	−0.7	0.8
I591D murine	−0.6	0.8

a Calculated with respect to the closest structure in the PDB (1U2C).

To validate the computational models involved in the current study, multiple approaches were employed. Firstly, PROCHECK was used to check the stereochemistry quality and structural feature, comparing the geometry of the residues in a given protein structure with the stereochemical parameters derived from crystal or NMR structures [29]. The PROCHECK result shows that 83.6% (wild-type) and 84.2% (V567D) residues are located in favored core regions, 13.0% (wild-type) and 12.3% (V567D) in allowed regions, 2.1% (wild-type) and 3.4% (V567D) in generously allowed regions and 1.4% (wild-type) and 0.0% (V567D) in disallowed regions. For a good quality model, it is expected that the residues located in the most favorable and additional allowed regions should be more than 90%, which is the case for the computational structures of both wild-type and V567D α-DG C-terminal regions [47]. Residues Lys556 and Arg614 of wild-type were in disallowed region. It is worthwhile to remember here that both these residues are not located within secondary structure elements. Secondly, to further check the global quality of the computational model, the program VERIFY3D was used to analyze the compatibility of the residues with their environment [30]. Residues with a positive score should be considered reliable. In the current case, VERIFY3D result shows that 96.4% (wild-type) and 92.1% (V567D) of the residues in our computational models has an averaged 3D–1D positive score, suggesting that the model has overall self-consistency in terms of sequence-structure compatibility. The 3D–1D profile score dips below 0 at six points in the wild-type model (from Val569 to Gly573 and Ala590) and nine points in the V567D model (from Val569 to Lys577) all belonging to the unstructured region connecting the Ig-like domain with the coil-strand-coil region. ProSA (Protein Structure Analysis) [31] was adopted to further check the quality of the generated models. The Z-scores, a parameter describing the overall model quality, are predicted to be −4.5 and −4.6 for wild-type and V567D structure model, respectively. Both values are within the range of Z-scores found for native proteins of similar size, indicating that the overall quality of our model is high. A summary of the results obtained from these programs, indicating that the residues in the model are placed in a very good overall configuration, is reported in Table 2.

**Table 2:** Evaluation of I-TASSER models by using PROCHECK, ProSA-Web and VERIFY3D protein structure evaluation tools.

α-DG	PROCHECK					ProSA
	Ramachandran plot statistics (%)^a^				VERIFY3D	
	Core	Allowed	General	Disallowed	Compatibiliy score (%)^b^	z-score
Wild-type zebrafish	83.6	13.0	2.1	1.4	96.4	−4.5
V567D zebrafish	84.2	12.3	3.4	-	92.1	−4.6
Wild-type murine^c^	94.5	4.8	-	-	100.0	−5.4
I591D murine	87.6	11.0	1.4	-	99.4	−4.5

a Ramachandran plot qualities show the percentage (%) of the residues belonging to the favoured (core), additionally allowed (allowed), generously allowed (general), disallowed region of the plot.

b Percentage (%) of the residues with compatibility score above zero.

c Data from De Rosa et al [17].

### *In silico* modelling of murine I591D α-DG C-terminal region

Primary sequence and secondary structure prediction by I-TASSER of murine wild-type and I591D α-DG C-terminal region are shown in Figure S1. Analogously to the zebrafish DG, only β-strands and coils were predicted in the secondary structure of the region spanning residues 500–600, whereas coils, α helices and strands were found in the extreme C-terminus. The I-TASSER server used the crystal structure of the murine α-DG N-terminal domain (PDB 1U2C [19]) and of mouse E-cadherin ectodomain (PDB 3Q2V [48]) as template structures to assemble the model of the I591D C-terminal domain of murine α-DG. Statistical support for the predicted structural models is reported in Table 1. The two major criteria, the C-score and the TM-score, indicate reasonable models with very similar overall topology and a high degree of three-dimensional structure similarity. As expected, according to I-TASSER, the model of the I591D α-DG C-terminus is similar in structure to 1U2C (Cα RMSD = 1.73 Å) and consists of an Ig-like domain (residues 500–600) and a coil–helix–coil region (601–653). The structure and topology of the mutant murine Ig-like domain closely resemble those of the wild-type [17] and of the predicted zebrafish Ig-like (Fig. 2).

Analysis using PROCHECK [29] indicates excellent geometry with no residues in disallowed regions of the Ramachandran plot. 87.6%, 11.0% and 1.4% of the residues fall into the favored core, allowed, and generously allowed regions, respectively. For the most representative structure VERIFY3D and ProSA profiles also are indicative of a high quality model. In the VERIFY3D scan, the designed model shows that all the residues have an average 3D–1D positive score with the exception of Pro614, which is however located in the predicted random coil region in the extreme C-terminus. A ProSA Z-score of −4.5 also confirms the good quality of the model. Assessment of the three-dimensional model is summarized in Table 2.

### Molecular dynamics conformational flexibility and stability analysis

**Figure 3:**
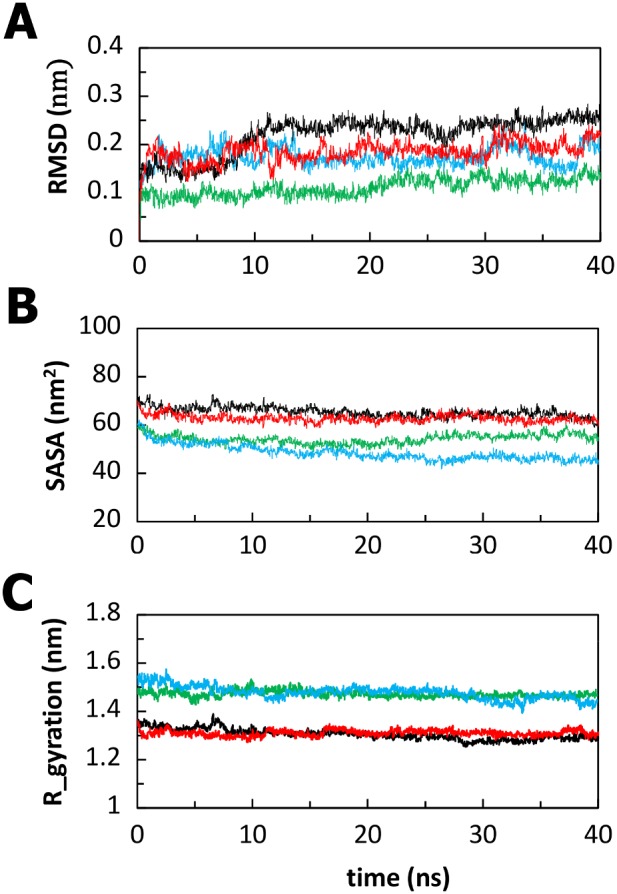
Evolution of the structural properties of the Ig-like domain belonging to the α-DG C-terminal region over time. Cα RMSD (panel A), Solvent Accessible Surface Area (panel B), and Radius of gyration (protein) (panel C) of the Ig-like domains of wild-type zebrafish (black), V567D zebrafish (red), wild-type murine (green) and I591D murine (light blue).

To check the stability of the simulations, the RMSDs of the Cα atoms with respect to the minimized starting structure, radii of gyration (Rg) of the protein and Solvent Accessible Surface Area (SASA) of protein were calculated and monitored over the course of simulations and are presented in Figure 3. Evaluation of the structural drift was provided by the analysis of the Cα atom RMSDs from the initial structures as a function of time. The RMSDs of the Ig-like domains through the 40 ns trajectory were computed with respect to their corresponding initial minimized structures (Fig. 3A). In all four cases, the RMSD shows convergence of the simulation within 40 ns (Fig. 3A). The wild-type murine protein presents the smallest deviation and adopted after 200 ps a stable conformation not so far from the initial one (0.1 nm), indicating that this system was very stable during the simulation. The RMSD of wild-type zebrafish simulation converges after 10 ns to a value around 0.24 nm, whereas in the mutants simulations RMSD increases sharply till 1 ns and reaches a value of 0.20 nm (zebrafish V567D) and 0.17 nm (murine I591D) remaining reasonably stable until the end of the simulation (Fig. 3A). Figure 3 demonstrates that for all structures, the RMSD remains stable around average values of 0.1–0.2 nm (Table 3) over a considerable time period (30 ns) of the later part of the trajectory. The SASA and Rg are related to (and give a global account of) the general tertiary structure of the protein. The curves of SASA_TOTAL_ indicate that the exposed areas (both hydrophobic and hydrophilic), for all the systems investigated, although slightly decreasing with the mutation (an effect more evident in the case of zebrafish), are stable during the entire simulations (Fig. 3B). As expected from the RMSD analysis, it is not possible to observe significant changes in the Rg during the simulations. The plot of Rg versus time is presented in Figure 3C. For both zebrafish and murine systems the curves do not differ significantly and maintain the lowest value of Rg around ∼1.3 nm (zebrafish) and ∼1.5 nm (murine), indicating that the compact conformation is largely preserved upon mutation (Fig. 3C). The time averaged structural properties are reported in Table 3.

**Table 3:** Time averaged structural properties calculated for wild-type and mutant Ig-like domains.

	zebrafish		murine	
	WT	V567D	WT	I591D
Cα-RMSD (nm)	0.24(0.02)	0.19(0.02)	0.12(0.02)	0.18(0.02)
SASAtotal (nm^2^)	66.14(2.07)	62.82(1.60)	53.32(1.98)	48.52(3.26)
Rg-protein (nm)	1.31(0.02)	1.31(0.01)	1.37(0.03)	1.47(0.01)

Standard deviations are given in parentheses.

**Figure 4:**
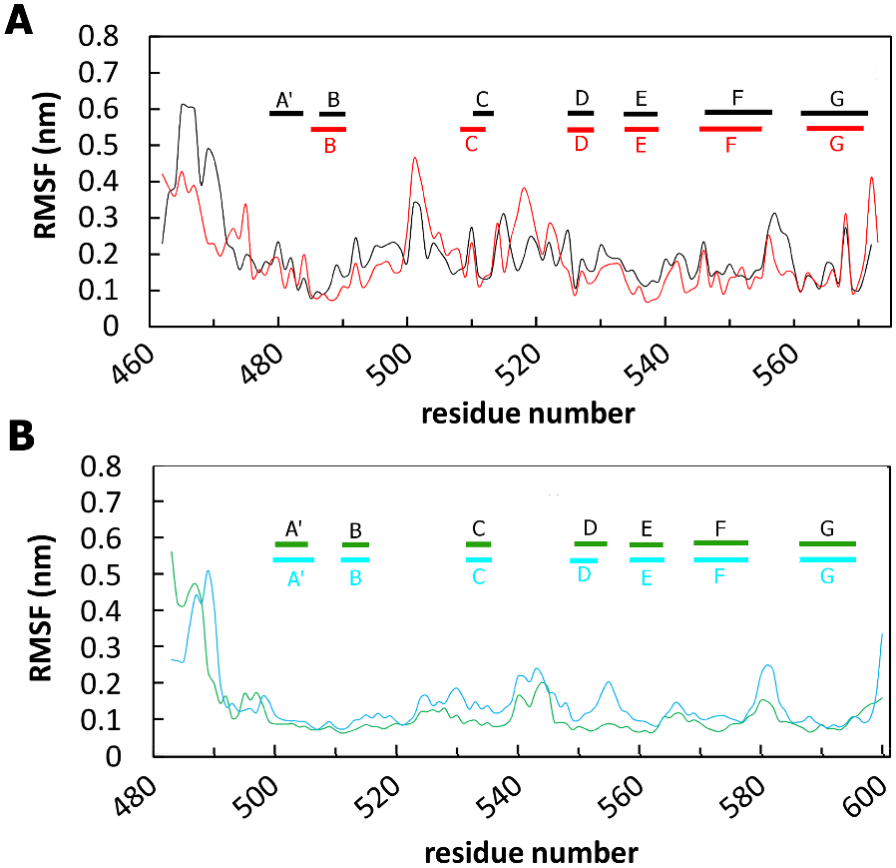
Cα-RMSF values averaged per each residue over the last 30 ns of MD trajectory. Wild-type (black) and V567D (red) zebrafish simulations are shown in panel A; wild-type (green) and I591D (light blue) murine simulations are shown in panel B. Only the protein region spanning the Ig-like domain is shown.

Interesting information comes from the root mean square fluctuations (RMSFs) of each amino acid (Fig. 4), which highlights the flexible regions of the systems. RMSFs values higher than 0.25 nm are characteristic of amino acid residues belonging to flexible regions. For all the systems analysed, the loop at the N-terminus and the loops between the β-strands displayed RMSFs values which are typical of flexible regions, while the regular secondary structure regions showed small fluctuations during the simulations. In the zebrafish DG, the most pronounced Cα-RMSF differences between the wild-type and the mutant occur for residues 500–502 and 517–519, which belong to the long loops connecting strands B and C, and C and D, respectively (Fig. 4A). In these two regions the V567D Cα-RMSF is ∼1.5 and ∼2 times larger than that of the wild-type, respectively. The RMSFs values observed for murine DG exhibit more or less similarly distributed fluctuations. Most of the residues, which belong to the loops connecting strands C and D, D and E, F and G, become highly mobile upon mutation (Fig. 4B).

**Figure 5:**
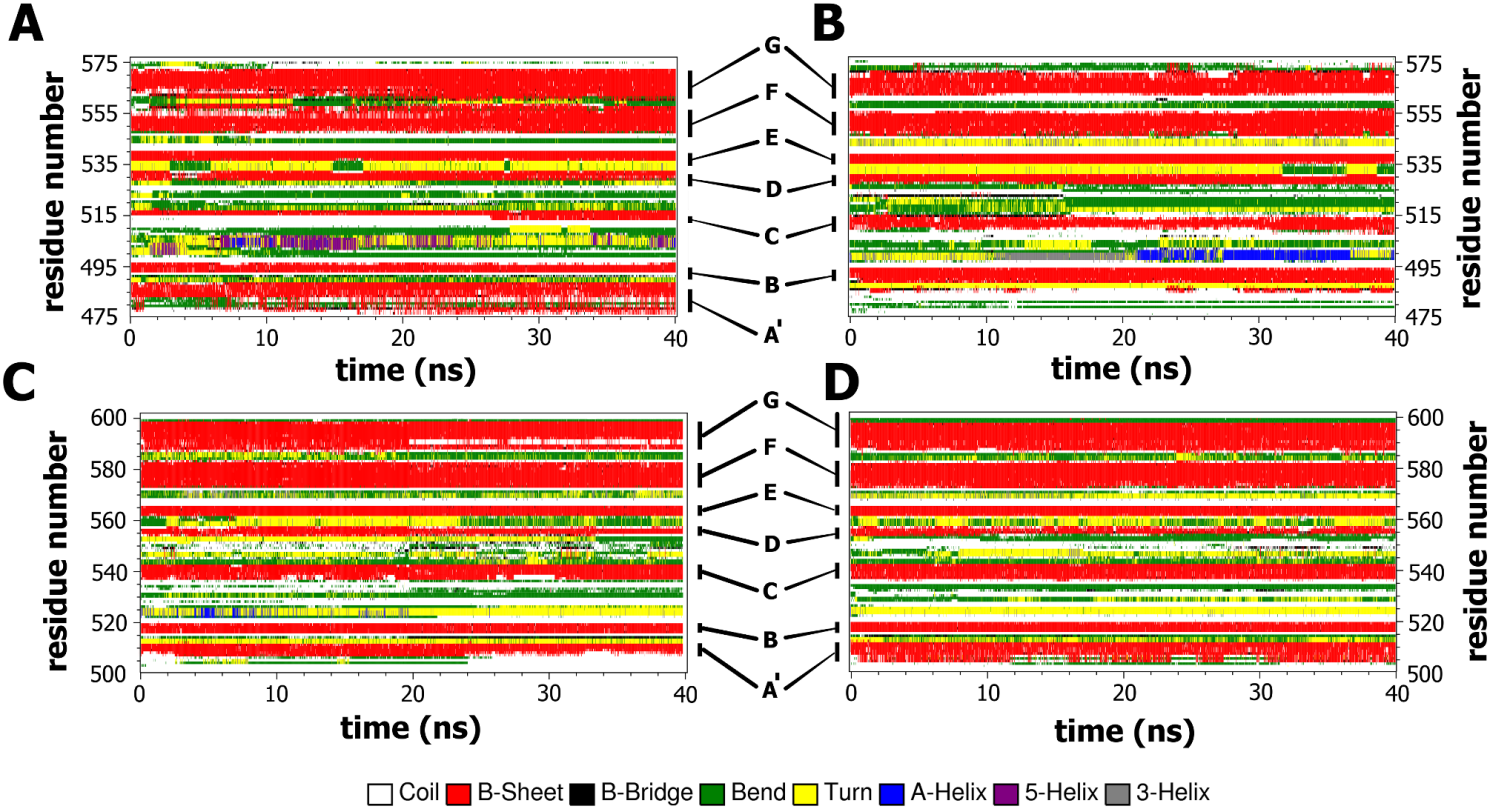
Time evolution of the secondary structural elements along the MD simulation generated by DSSP. Wild-type zebrafish (panel A); V567D zebrafish (panel B); wild-type murine (panel C); I591D murine (panel D). The X-axis represents the MD trajectory time (in ns), while the residue numbers are shown on the Y-axis. Only the protein region spanning the Ig-like domain is shown.

We then tried to examine whether the mutation induced any changes in the secondary structural elements during the simulations. Figure 5 shows the classification of the four trajectories in terms of secondary-structure elements obtained by the software tool DSSP [39], whose plots enable a local structural analysis complementing the above characterization of the dynamics. The stability of the secondary structures was examined during the entire period (40 ns) (Fig. 5). Interestingly, among the four simulated systems, the V567D zebrafish shows a strong disorganization of strand A′ (Fig. 5B), a phenomenon not observed in the other models, whose secondary structure elements appeared very stable during the entire MD simulations. Examination of Figure 5 shows that the main features of the β-sheets structure are largely retained, i.e., the strands A′, G, F, C, and the others B, E, D are preserved throughout the 40-ns simulation for wild-type zebrafish (Fig. 5A), wild-type murine (Fig. 5C) and I591D murine α-DG (Fig. 5D). By contrast, at 0.1 ns of the V567D simulation, most of the A′ strand unfolds and is converted into loop giving rise to a long flexible region at the N-terminus of the domain (Fig. 5B). Large scale fluctuations from helical to bend or turn structures at the long loop connecting strands B and C are observed in all the systems (Fig. 5).

### Effects of V567D and I591D substitutions on the stability of the Ig-fold

**Figure 6:**
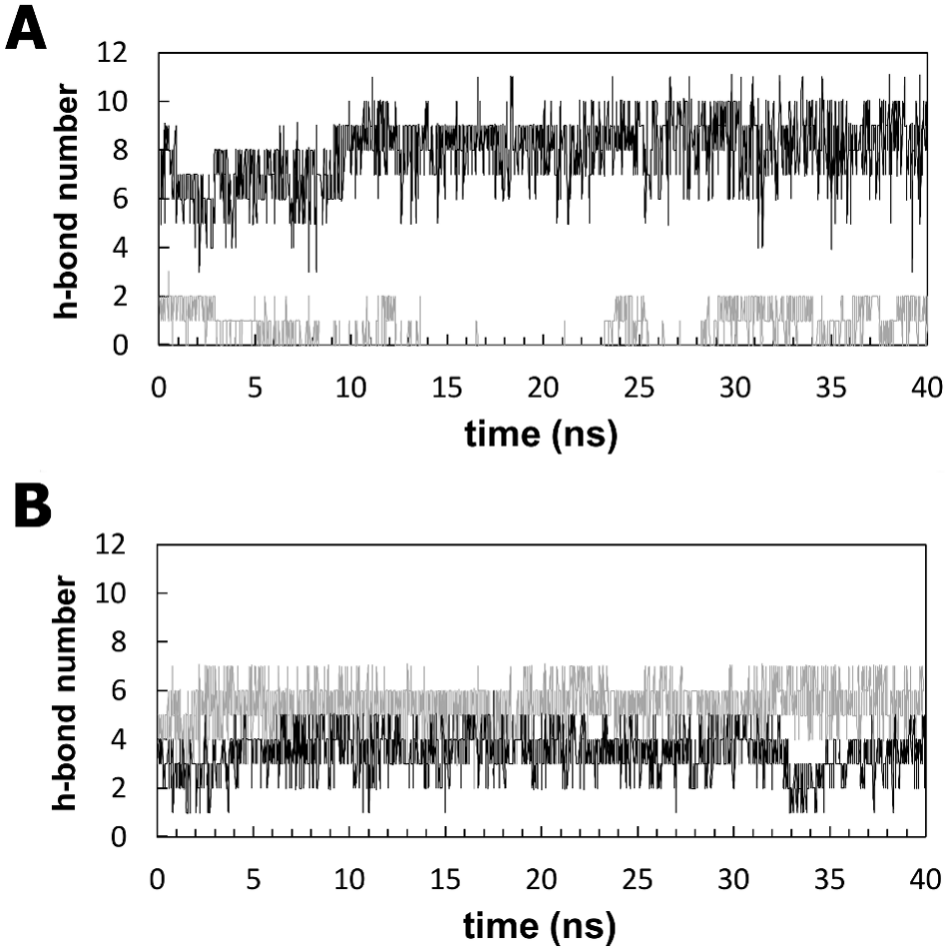
Backbone hydrogen bonds along the simulation trajectories for the four models. Shown is the number of backbone hydrogen bonds formed between the A′ and the G strands of zebrafish (panel A) and murine (panel B) α-DG Ig-like domains. The black and gray lines show the trajectories for wild-type and mutant systems, respectively.

**Figure 7:**
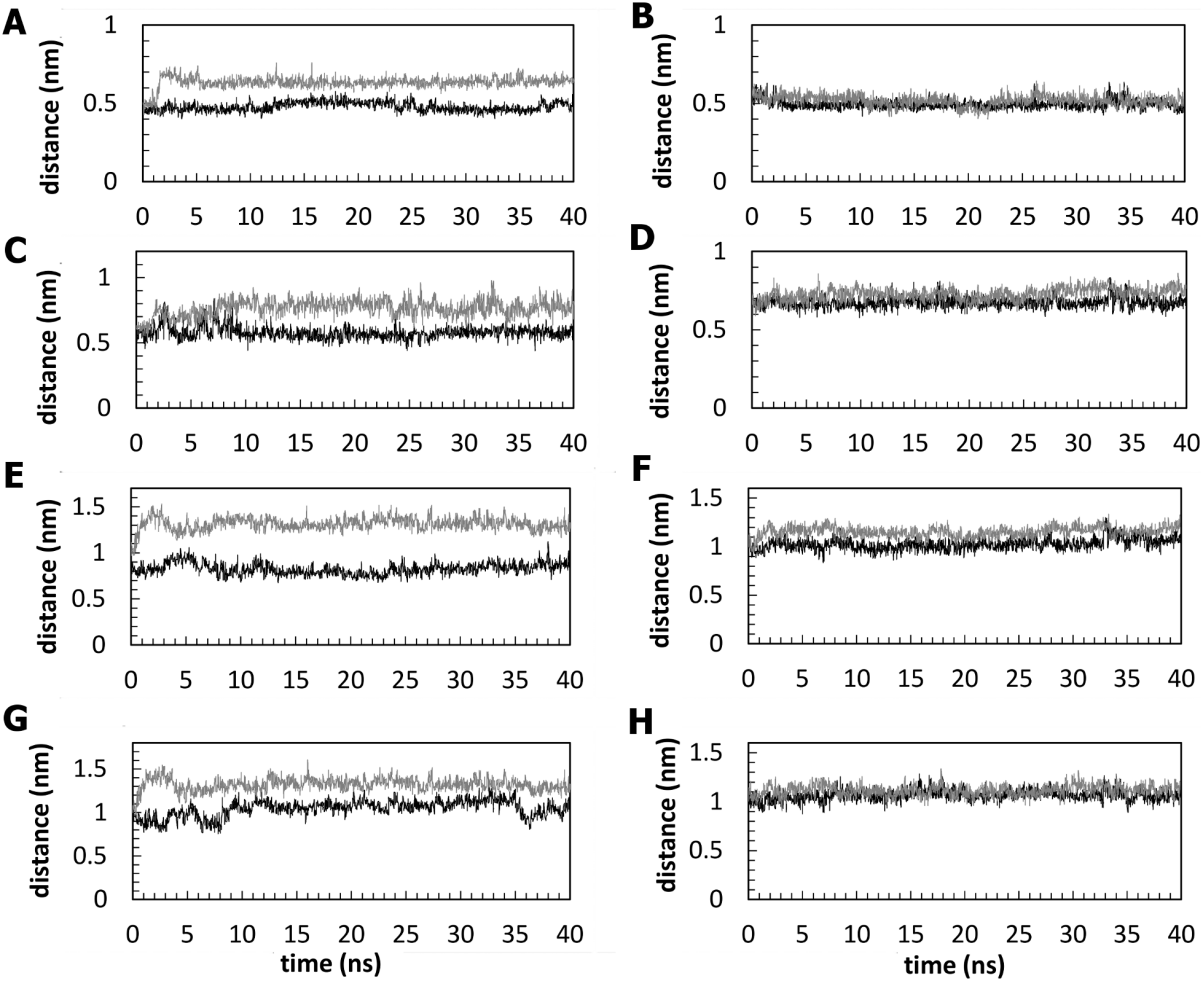
Distance analysis between the A′–G and the B–G strands. Time evolution of the distances between Cα atoms of zebrafish residue pairs 481–567 (panel A), 483–567 (panel C), 489–567 (panel E) and 491–567 (panel G) and of murine residue pairs 504–591 (panel B), 506–591 (panel D), 512–591 (panel F) and 514–591 (panel H). The black and gray lines show the trajectories for wild-type and mutant systems, respectively.

The Ig-like domain is stabilized by hydrophobic core interactions between the two β-sheets and by the hydrogen bonds between the β-strands [39], [49]. Interfering with any of the residues in the sheet by a mutation may lead to a discontinuity in the hydrogen bonding pattern, which is characteristic of the Ig-like domains. This may enhance the conformational flexibility of the mutated residue side chain, which could disrupt the natural bonding of neighbours and might result in loss of secondary structural elements [50], [51]. The external strands A′ and G present geometrical distortions known as β-bulges, as found in some Ig molecules [52], which lead to an imperfect general H-bond network. However, examination of the hydrogen bond patterns involving the β-strands A′–G reveals significant differences among the simulated systems (Fig. 6). Figure 6A shows that the backbone hydrogen bonds formed between the strands A′ and G, where the mutation is located, are stable in zebrafish wild-type but are disrupted in the zebrafish V567D mutant, resulting in a significant separation between the two strands in the β-sheet. By contrast, the corresponding backbone hydrogen bonds in murine DG were not noticeably affected by the I591D mutation (Fig. 6B). The changes in the hydrogen bond pattern observed in zebrafish DG are closely related to the disruption of the native hydrophobic contacts. Val567 residue, located on the G strand, interacts with a number of hydrophobic residues nearby and the strongest interactions are observed with Val481(β-strand A′), Ala483(β-strand A′), Phe489 (β-strand B) and Val491 (β-strand B). Significantly, unlike Val567, the acidic Asp567 residue of mutant DG maintains its side chain exposed to the solvent over the simulation time. Analysis of the MD trajectories shows that the hydrophobic contacts involving the 567 position remain relatively stable in the wild-type with the Val567 residue continuously interacting with residues Val481, Phe489, Ala483 and Val491 whereas they are disrupted upon mutation. This results in a significant disorganization of the A′ strand and in a widening of the cleft between the sheets of the Ig-like domain. This effect is not observed in murine α-DG, in which the hydrophobic contacts established by Ile591 with Val504, Ala506, Phe512 and Val514 are well preserved after the mutation. The Cα-Cα distances between the above-mentioned residues are reported in Figure 7 for both, zebrafish (A, C, E and G panels) and murine (B, D, F, and H panels) protein models, in comparison with their mutated counterpart. Panels A, C, E and G highlight the separation between A′–G (Fig. 7A, C) and B-G (Fig. 7E, G) strands. Notably, the large differences observed between Cα of 489, 491 (strand B) and 567 (strand A′) positions (Fig. 7E, G) indicate the separation between the two sheets of the β-sandwich (Fig. 2). In the case of murine α-DG the I591D replacement produces no effect on the corresponding distances between A′–G (Fig. 7B, D) strands and very little effects on the separation between the sheets (Fig. 7F, H).

**Figure 8:**
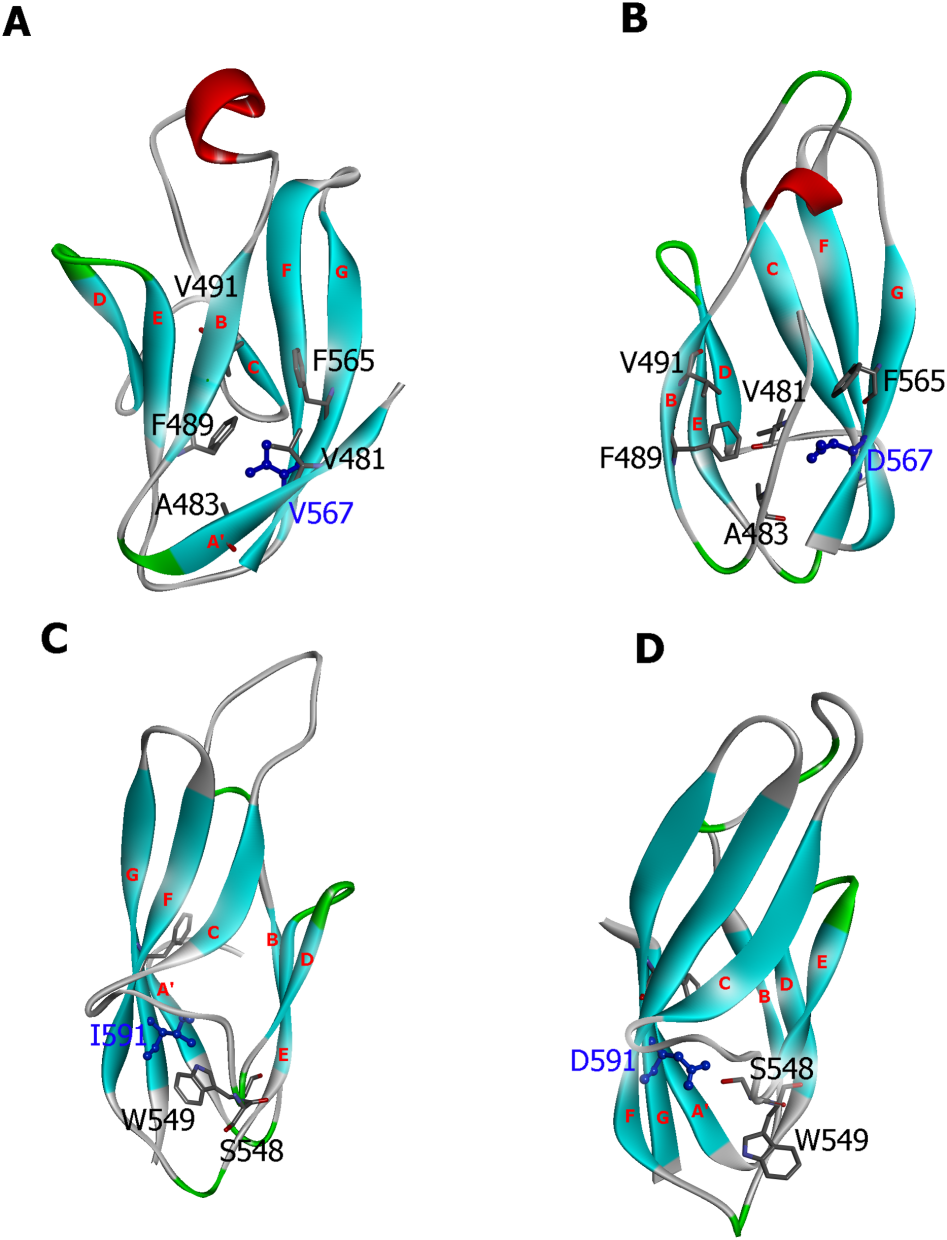
Structural comparison of the predicted wild-type and mutant α-DG Ig-like domains. The wild-type zebrafish (Panel A), the zebrafish V567 mutant (Panel B), the wild-type murine (Panel C) and the murine I591D mutant (Panel D) models are shown using their corresponding average structure of the last 25 ns simulation. The location of the residues described in the current study and strands A′, B, C, D, E, F, and G are also labeled.

We also examined the structural conformations obtained from the MD simulations of the wild-type and the mutant α-DG to better evaluate the conformational change in the mutant protein upon the amino acid replacement. Structural comparison between the average structures generated from the last 25 ns MD trajectory is shown in Figure 8. TM-score can be used as an approximate but quantitative criterion for protein topology classification, i.e. protein pairs with a TM-score>0.5 are mostly in the same fold, while those with a TM-score<0.5 are mainly not in the same fold [53], [54]. A TM-score of 0.55 and 0.71 were calculated for zebrafish and murine average structures, respectively, indicative of a low similarity between the wild-type and the mutant zebrafish protein. As shown in Figure 8 the V567D substitution causes the unfolding of the A strand and the G strand pulling away from the β-sheet (Fig. 8A, B). As a result, the two β-sheets slide away from each other and the average distance between the center of mass of B and G strands increases from 10.3 Å (wild-type) to 14.8 Å (V567D) (Fig. 8A, B). The extent of the mutation-induced structural rearrangement can also be seen from the changes in the solvent exposure of the groups interacting with the mutation site. The segment that becomes more exposed to the solvent upon V567D mutation is the B-strand (36% SASA increase). Notably, we observed a significant increase in SASA of Val491 whose value is 14 Å^2^ in wild-type and 32 Å^2^ in V567D, in agreement with the analysis of Halaby and coworkers [49], who calculated, for amino acids of the internal strand B with side chains pointing towards the interior of the protein, a SASA value <20 Å^2^ and for amino acids with side chains pointing towards the exterior of the protein SASA values between 20 Å^2^ and 50 Å^2^. In the wild-type and I591D murine average structures, we focused on the D-strand which appears as the most affected region upon mutation. The strong hydrophobic interactions involving Ile591 and Trp549 (D strand) in wild-type murine α-DG are shown in Figure 8C. Notably, the SASA analysis shows that there is a drastic increase in SASA for Trp549, from 18 Å^2^ (wild-type) to 45 Å^2^ (I591D), values that are in reasonable agreement with the SASA values expected for residues of an external strand as the strand D [49]. These changes are possibly triggered by the amino acid replacement at Ile591 position, which affected the normal interactions between Ile591 and Trp549. Effectively, in the mutant murine DG the Asp591 side chain forms an hydrogen bond with Ser548 and this results in the movement of the indole ring belonging to Trp549 towards the solvent (Fig. 8D), an event that might induce a significant destabilization [55]. On the whole, the establishment of new contacts that remain stable during the simulation suggests that the Ig-like domain of murine α-DG should not display an impaired stability when Ile591 is replaced by Asp. Nevertheless, possible structural-functional consequences derived from the observed structural rearrangement cannot be ruled out as indicated by our analysis of the recombinant protein carrying the I591D mutation (see below).

### Preparation of the recombinant murine α-DG(485–630)I591D mutant

In order to test the stability of the mutant α-DG C-terminal domain, namely α-DG(485–630)I591D, we prepared this construct using our consolidated prokaryotic expression system (*E. coli*) that we have previously used for analyzing a plethora of murine domains of DG [21].

**Figure 9:**
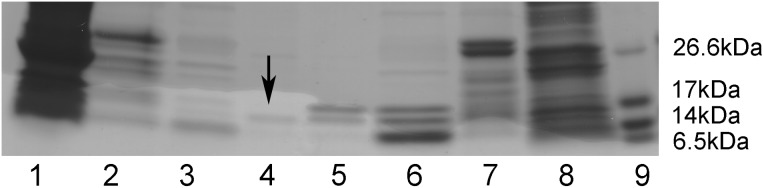
Recombinant expression of α-DG(485–630)I591D. The recombinant murine mutant α-DG(485–630)I591D as well as its wild-type counterpart were purified by affinity chromatography. The fractions collected after each purification step were run on the same SDS-PAGE: lane 1: total protein extract from *E. coli* cells expressing 6xHis-Trx-α-DG(485–630)I591D; lane 2: purified 6xHis-Trx-α-DG(485–630)I591D; lane 3: 6xHis-Trx-α-DG(485–630)I591D upon thrombin cleavage; lane 4: purified α-DG(485–630)I591D (arrow); lane 5: purified wild-type α-DG(485–630); lane 6: 6xHis-Trx-α-DG(485–630) upon thrombin cleavage; lane 7: purified 6xHis-Trx-α-DG(485–630); lane 8: total protein extract from *E. coli* cells expressing wild-type 6xHis-Trx-α-DG(485–630); lane 9: protein markers.

The recombinant mutant α-DG(485–630)I591D, expressed as a fusion protein conjugated with six N-terminal histidine residues and the thioredoxin (Trx), was purified by affinity chromatography using a nickel nitrilotriacetate resin. After thrombin cleavage to separate α-DG(485–630)I591D from its fusion partner, the protein was submitted to a further affinity chromatography step to remove the fusion partner from the solution. A similar protocol was applied to the wild-type protein in order to compare the stability of the two proteins (Fig. 9). Any attempt made to further purify the I591D mutant was unsuccessful because of its high propensity to degradation. Figure 9 shows an SDS-PAGE, in which protein samples at different stages of the purification protocol were analyzed. The purified protein, compared to its wild-type counterpart, displays a faint band corresponding to the lower degraded band observed in the wild-type, while no signal corresponding to the full-length protein can be observed. At the present stage, due to this pronounced unstable behavior, it is actually impossible to collect significant amounts of the I591D variant to be employed for its biochemical characterization.

### Analysis of the expression of the mutant I591D in transfected 293-Ebna cells

We further assessed the effects of the I591D mutation *in vitro*, with respect to expression and post-translational processing of DG. To this end, we transiently expressed the full-length wild-type and I591D DG proteins in 293-Ebna cells using two DNA constructs carrying a myc-tag inserted at the position K498 of the C-terminal domain of α-DG and cloned in a pEGFP vector [40].

**Figure 10:**
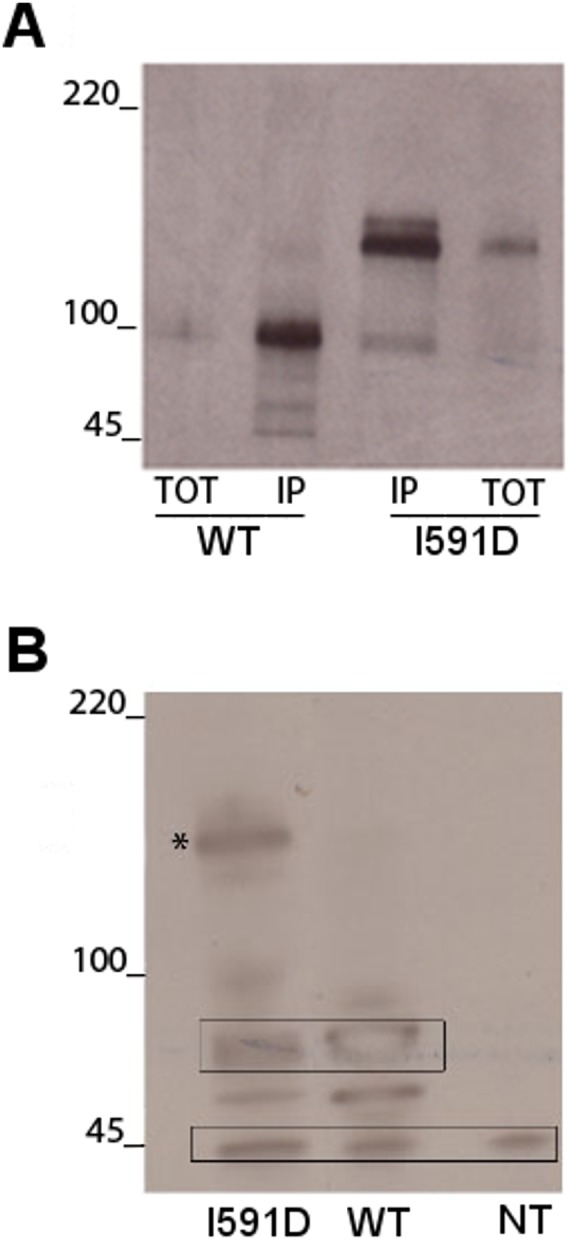
I591D mutation partially prevents the post-translational cleavage of murine DG precursor. 293-Ebna cells were transfected with the wild-type or the mutated I591D constructs both carrying a myc-tag within the C-terminal domain of α-DG and cloned into the pEGFP vector. A) Immunoprecipitation with an anti-myc-antibody of wild-type and I591D α-DGs. In cells transfected with wild-type DG the slightly broad band detected at 100 kDa (TOT), that is further enriched upon immunoprecipitation (IP), corresponds to the mature α-DG. In cells transfected with the I591D mutant an additional and prominent band is detected at 160 kDa corresponding to the uncleaved DG precursor. B) Western blot of total protein extracts probed with an anti β-DG antibody. The wild-type construct displays a single band at 60 kDa corresponding to the mature β-DG-GFP, while I591D shows an additional band at 160 kDa corresponding to the unprocessed DG precursor (asterisk). The band at 45 kDa represents the endogenous β-DG.

Interestingly, the mutation does not prevent or downregulate the expression of DG compared to the wild-type, however the structural rearrangements occurring in I591D partially impair the post-translational cleavage of the mutated DG precursor. In fact, an additional band at about 160 kDa is detected in Western blot using anti β-DG or anti-myc antibodies (Fig. 10 A and B). It was already shown that mutations that affect the stability of the DG precursor, such as the disruption of disulfide bridge within the extracellular domain of β-DG or the perturbation of the interaction between the two subunits, strongly influence its post-translational cleavage and plasma membrane targeting [21], [56], [57].

## Conclusions

Understanding the molecular consequences of the mutations that cause human genetic diseases remains an important research challenge. Missense mutations may have diverse structural and functional effects on proteins, ranging from changes in the folding pathway that may affect their overall stability to alterations in their ligand binding properties. Computational methods have been widely used to assess the structural effects of genetic variants and to investigate the detailed mechanisms underlying the pathogenicity of missense mutations.

Given our previous biochemical and computational work on the C-terminal region of α-DG [17], [21], it was of great interest for us that a recently discovered point mutation (c.1700T>A) in the gene *DAG1* of zebrafish, resulting in the V567D missense mutation, could induce a very strong destabilization of the protein eventually leading to the absence of protein and to a reduction of its mRNA levels [16].

In this study, the impact of the single amino acid substitution V567D on the stability of α-DG was evaluated combining a number of computational methods to improve prediction. Our findings provide new insights into the structural basis for the reported dramatic destabilization of zebrafish DG induced by the V567D mutation and gives a possible molecular explanation to understand how the homologous and topologically related I591D mutation in murine DG could also compromise the protein function. The presence of a hydrophobic residue in this position, such as a Val or Ile, is highly conserved within all the DG sequences so far analyzed [21]. Although belonging to an external strand (G), these residues are also involved in forming hydrophobic interactions with the internal core of the Ig-like domain. We have shown that Val567 residue plays a pivotal role in maintaining the hydrophobic core structure of the Ig-like domain. By a set of molecular dynamics simulations, in which the dynamics of wild-type and mutated DG were compared, evident signs of stability loss provoked by the V567D mutation were observed in the number of hydrogen bonds, hydrophobic contacts and inter-strand packing distances between the β-sheets of the Ig-fold. The local perturbation at the G-strand may function as a nucleation site for the unfolding of the protein and account for the experimentally observed destabilization [16]. We can hypothesize that in zebrafish the severe perturbation of the central hydrophobic core structure of the Ig folded domain prevents the correct folding of the DG precursor impairing its entire maturation and targeting pathway, in line with the accepted idea that the central core of the Ig fold serves as a scaffold for the presentation of sites involved in molecular recognition, cell adhesion, and ligand binding [58], [59].

In the case of I591D murine DG, important conformational changes were found to occur within a short time, suggesting potential changes from the native structural properties within this protein region. Although we found that the I591D mutation is not likely to change the overall stability or dynamics of the entire protein region and in particular of the Ig-like domain, however, it brings about a significant local perturbation featuring the exposure of Trp549 towards the solvent. Namely, the D strand dynamics varies in a way that may still suggest a disturbance to the structural integrity of the domain. This event may account for the reduced expression level and stability of the recombinant domain expressed in *E. coli* and the alteration of the maturation pathway observed in the transfected eukaryotic cells. On the whole, theoretical and experimental findings demonstrated that the I591D mutation can affect several biophysical characteristics simultaneously and it may therefore lead to a certain degree of instability as indicated by the high propensity to degradation displayed by the recombinant α-DG C-terminal domain (see Fig. 9) and by the altered maturation pathway of DG, observed in our experiments using transfected cells (see Fig. 10).

The reduced affinity displayed by hypoglycosylated DG towards laminin is believed to represent the major molecular clue behind a number of secondary dystroglycanopathies [60], while much less is currently known on the molecular mechanism behind the two known primary dystroglycanopathies [6], [7]. It is interesting to note that the V567D/I591D mutation affects a domain of α-DG which is not extensively glycosylated. Only a few Thr residues within the loop interconnecting B and C strands were reported as GalNAc glycosylation sites and in particular the G strand, to which V567D/I591D belongs, was found unglycosylated [61]. Although we believe that the important role of this topological position within the G strand of the Ig-like domain of the C-terminal region of vertebrate dystroglycans is fully confirmed, we also believe that our analysis can be considered particularly interesting and innovative in the dystroglycan field since it is showing that even if the two orthologous proteins are highly conserved, the zebrafish background and the murine one have some obvious structural differences that in the future may be useful to define some species-specific different functional behaviours. In order to enlarge our knowledge on primary dystroglycanopathies, in the next future it will be more and more important to consider that also mutations affecting folding, stability and maturation of the DG precursor can lead to severe neuromuscular conditions as well as those affecting DG glycosylation’s shell. Our study reinforces the notion of the importance of a combined computational and biochemical approach for the study of complex diseases such as dystroglycanopathies.

## Supporting Information

Figure S1 **Primary sequences and secondary structure prediction by I-TASSER.** Prediction of the secondary structure of the zebrafish wild-type (panel A), zebrafish V567D (panel B), murine wild-type (panel C) and murine V591D (panel D) α-DG C-terminal regions. Strands (S), α-helices (H) and coils (–), as predicted by I-TASSER, are aligned with the corresponding amino acid together with the confidence score. The mutation point is underlined.
(TIF)

Figure S2 **Evolution of the average structural properties for the three simulations of the Ig-like domain belonging to the α-DG C-terminal region over time.** Cα RMSD (panel A), Solvent Accessible Surface Area (panel B), and Radius of gyration (protein) (panel C) of the Ig-like domains of wild-type zebrafish (black), V567D zebrafish (red), wild-type murine (green) and I591D murine (light blue).
(TIFF)

Figure S3 **Average Cα-rms fluctuations per residue for the three simulations.** Cα-RMSFs were calculated relative to the average structure over the last 30 ns of all three wild-type (black) and V567D (red) zebrafish simulations (panel A) and wild-type (green) and I591D (light blue) murine simulations (panel B). Only the protein region spanning the Ig-like domain is shown.
(TIFF)

Figure S4 **Time evolution of the secondary structural elements, along the three independent MD simulations, generated by DSSP.** Wild-type zebrafish (panel A); V567D zebrafish (panel B); wild-type murine (panel C); I591D murine (panel D). The X-axis represents the MD trajectory time (in ns), while the residue numbers are shown on the Y-axis. Only the protein region spanning the Ig-like domain is shown.
(TIFF)

Figure S5 **Backbone hydrogen bonds along the simulation trajectories for the four models.** The average numbers of total backbone hydrogen bonds formed between the A′ and the G strands of zebrafish (panel A) and murine (panel B) α-DG Ig-like domains are plotted. The black and gray lines show the trajectories for wild-type and mutant systems, respectively.
(TIFF)

Figure S6 **Distance analysis between the A**′**–G and the B-G strands.** Time evolution of the average distances, for the three simulations, between Cα atoms of zebrafish residue pairs 481–567 (panel A), 483–567 (panel C), 489–567 (panel E) and 491–567 (panel G) and of murine residue pairs 504–591 (panel B), 506–591 (panel D), 512–591 (panel F) and 514–591 (panel H). The black and gray lines show the trajectories for wild-type and mutant systems, respectively.
(TIFF)
