# Supplementary figures and images for: Insights from Molecular Dynamics Simulations: Structural Basis for the V567D Mutation-Induced Instability of Zebrafish Alpha-Dystroglycan and Comparison with the Murine Model
Source: PLoS One. 2014 Jul 31;9(7):e103866. doi: 10.1371/journal.pone.0103866 (PMC4117597; doi:10.1371/journal.pone.0103866)

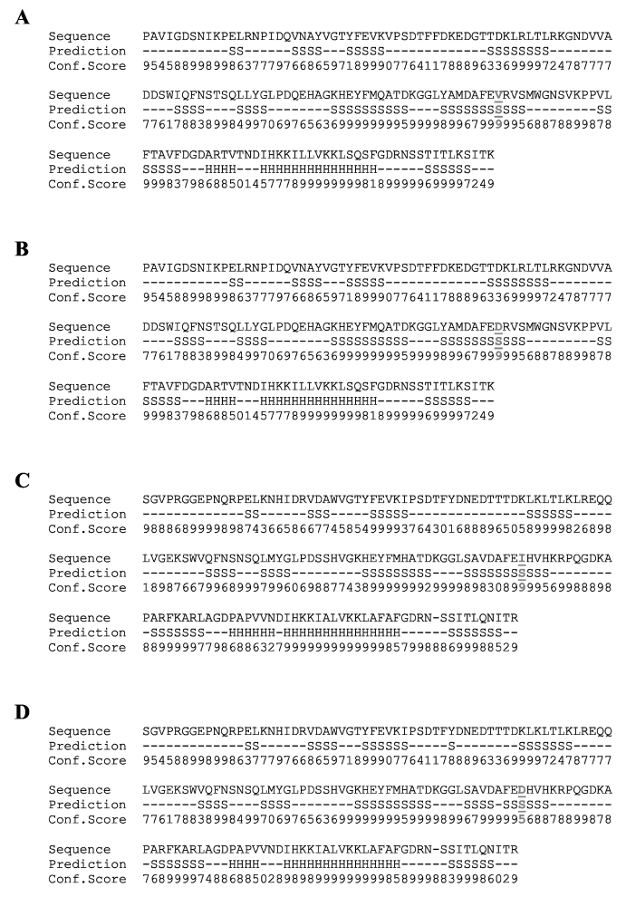

Supplement: Figure S1 — Primary sequences and secondary structure prediction by I-TASSER. Prediction of the secondary structure of the zebrafish wild-type (panel A), zebrafish V567D (panel B), murine wild-type (panel C) and murine V591D (panel D) α-DG C-terminal regions. Strands (S), α-helices (H) and coils (–), as predicted by I-TASSER, are aligned with the corresponding amino acid together with the confidence score. The mutation point is underlined. (TIF) [file pone.0103866.s001.tif]

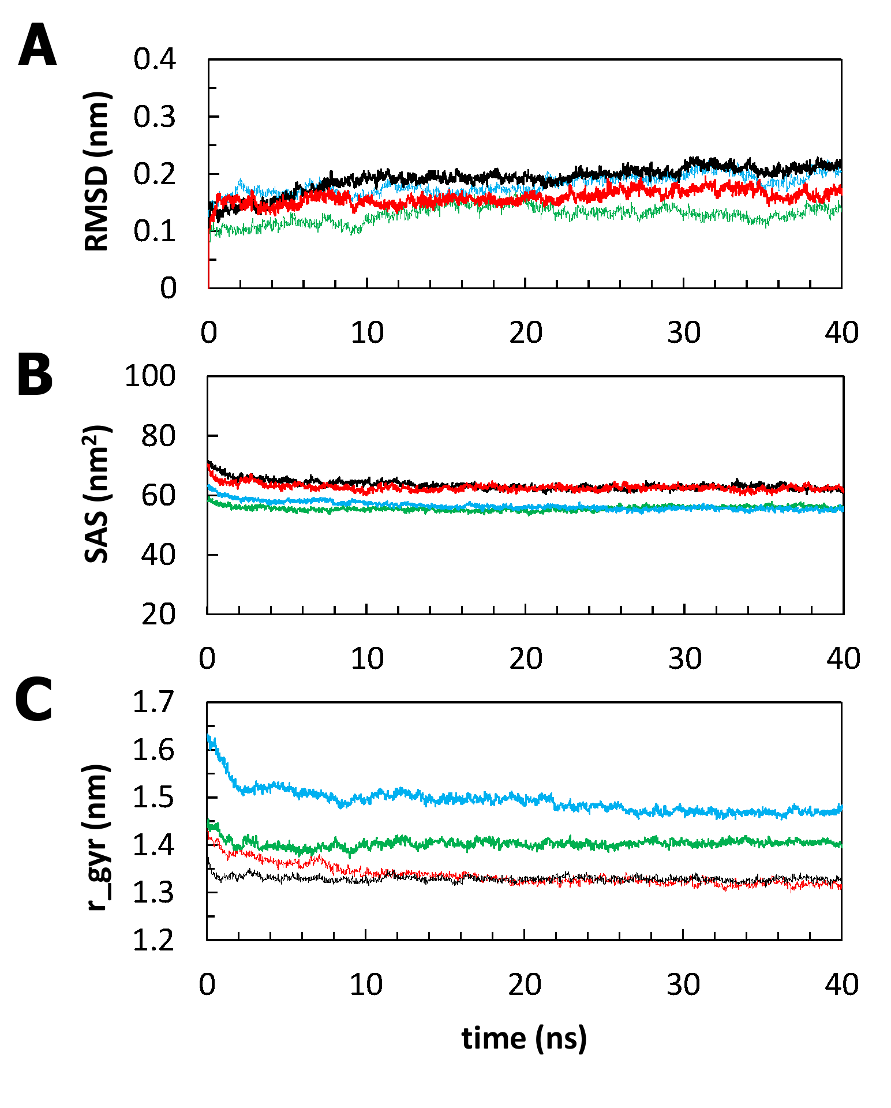

Supplement: Figure S2 — Evolution of the average structural properties for the three simulations of the Ig-like domain belonging to the α-DG C-terminal region over time. Cα RMSD (panel A), Solvent Accessible Surface Area (panel B), and Radius of gyration (protein) (panel C) of the Ig-like domains of wild-type zebrafish (black), V567D zebrafish (red), wild-type murine (green) and I591D murine (light blue). (TIFF) [file pone.0103866.s002.tiff]

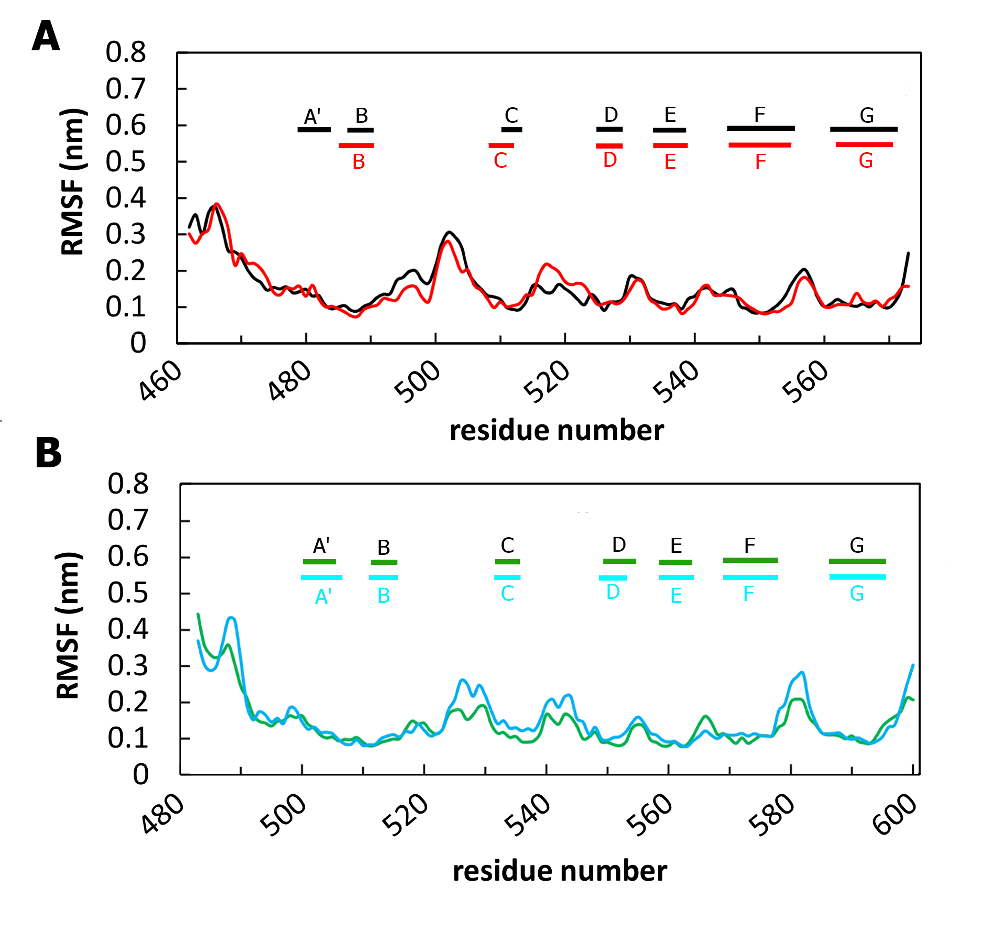

Supplement: Figure S3 — Average Cα-rms fluctuations per residue for the three simulations. Cα-RMSFs were calculated relative to the average structure over the last 30 ns of all three wild-type (black) and V567D (red) zebrafish simulations (panel A) and wild-type (green) and I591D (light blue) murine simulations (panel B). Only the protein region spanning the Ig-like domain is shown. (TIFF) [file pone.0103866.s003.tiff]

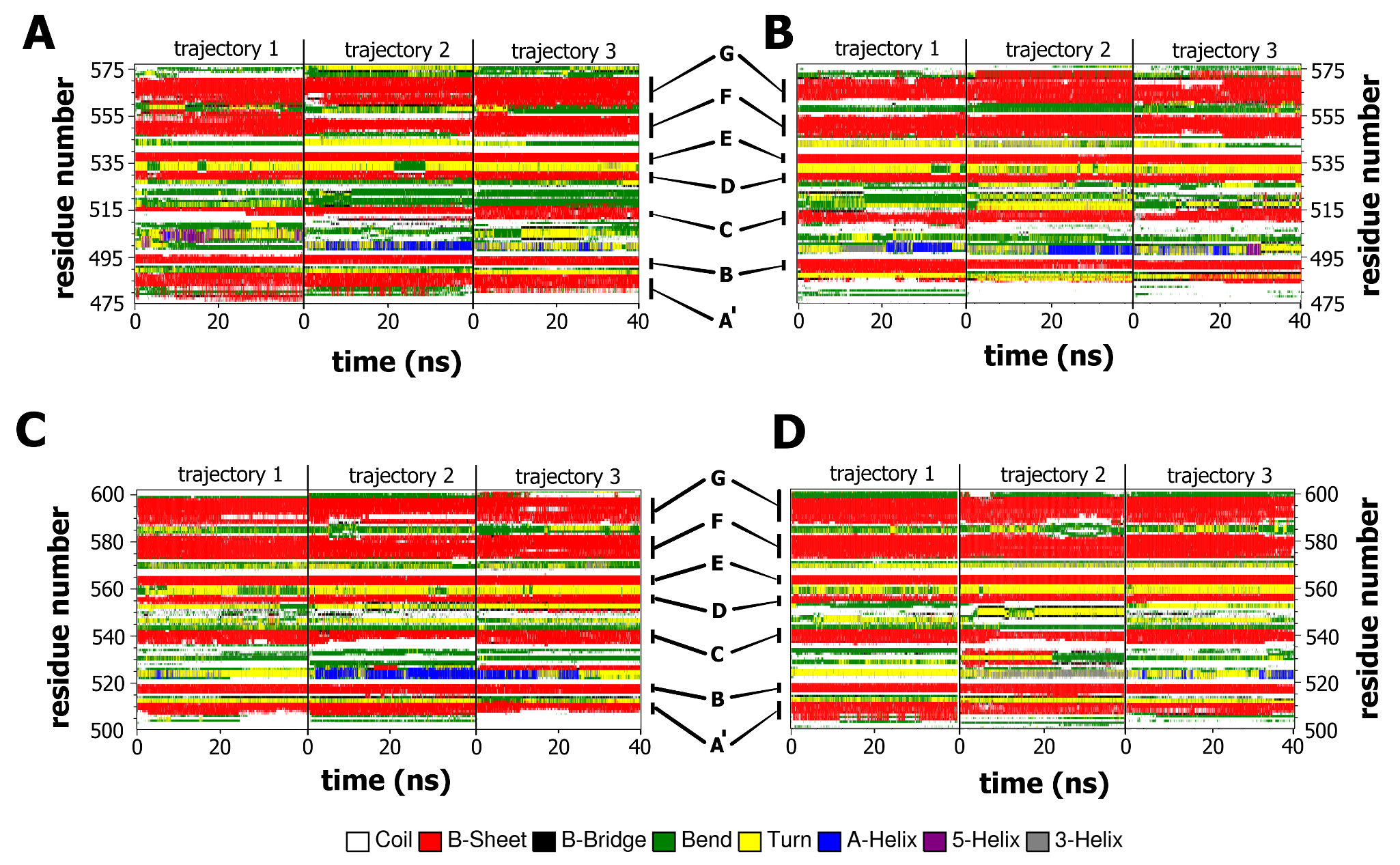

Supplement: Figure S4 — Time evolution of the secondary structural elements, along the three independent MD simulations, generated by DSSP. Wild-type zebrafish (panel A); V567D zebrafish (panel B); wild-type murine (panel C); I591D murine (panel D). The X-axis represents the MD trajectory time (in ns), while the residue numbers are shown on the Y-axis. Only the protein region spanning the Ig-like domain is shown. (TIFF) [file pone.0103866.s004.tiff]
